# How Are Deciduous Teeth Cast off

**Published:** 1889-11

**Authors:** Henry S. Chase

**Affiliations:** St. Louis.


ARTICLE VII.

### HOW ARE DECIDUOUS TEETH CAST OFF.

HENRY S. CHASE, M. D., ST. LOUIS.

It is by a physiological process as simple and beautiful as that by which their growth, dentification and calcification take place.

It is admitted by all that *absorption* takes place. Whether this process is excited by impingement of the crown of the tooth or replacement on the roots of the temporary teeth or not, is a disputed question.

My own opinion was expressed on a former occasion, in the following words: "Absorption of the roots of temporary teeth takes place in the ratio of the advancement of the permanent teeth, in the process of eruption, independently of their topographical relations."

ABSORPTION is a vital process and cannot take place in a dead bone, or tooth. Those who deny vital action in calcified dentine, will have hard work to prove that absorption does take place. A dead bone or dead tooth has no more physiological relations to living tissues than has iron or wood.

Physiological processes can take place only in living tissues.

Absorption is a word of ambiguous meaning, in its histological sense, in reference to the removal of the roots of teeth. At least most men seem to have no definite idea of the manner in which it takes place.

Absorption of the nutrient elements by the intestinal villi, is a mere reception of those elements into the pores or open months of the columnar epithelium of those villi. Absorption of water by the skin is an endosmosis, an active osmotic action in the cell walls or membranes of the skin.

ABSORPTION OF DENTAL TISSUES in deciduous teeth is a *retrograde* physiological process.

It is a work done neither by the dental blood vessels, the gum, a "carneous body," or by any other tissue or organ; but it is a work performed by the tooth itself.

Modern discoveries in physiology have demonstrated that all the active processes of life take place in the *ultimate* anatomical organs, which are *cells*. I shall assume the reader is acquainted with cellular physiology, for without this knowledge an intelligent understanding of the beautiful and delicate process of growth, disintegration and decay must ever remain to him an unknown world.

### THE PROCESS OF ABSORPTION.

The first thing which we observe on the extraction of a milk incisor in which this work has commenced, is a shortening of the root. The calcareous matter is gone, and so are the distinctive dentinal and cemental tissues. There are neither dentine tubes nor bone corpuscles left in the bottom of the alveolus; but in their place a mass composed mostly of histological connective tissue. The peri-cementum or alveolar dental periosteum is the first to change itself, and also to initiate change in the osteoid root itself.

The process commences near the *end* of the root, tho it sometimes does higher up on its lateral surface. The dental artery which supplies the root remains intact after considerable progress has been made in the work of metamorphosis. The arterial radicals of the peri cementum enlarge, they carry a greater amount of blood than before; especially is this the case when the dental artery has been destroyed by impingement of the crown of the tooth of replacement again it. These arterial radicals lengthen also as the process of metamorphosis goes on, so that the vessels are always near the scene of action to carry food for the work, and also to remove effete materials.

Simultaneously with this work going on in the root, a change takes place in the dental pulp; the cavity in which it is situated enlarges in every direction, and particularly toward the distal portion of the crown, so that the horns of the pulp reach nearer the grinding surface of the crown than before. As this process proceeds, the pulp itself grows larger in corresponding directions, and keeps even pace with the enlargement of the cavity, so that its cells are always in contact and union with the unmetamorphosed portion.

As the root is more and more removed, the space which it occupied is filled with bone which has been differentiated from connective tissue. The bottom cells are changed to bone corpuscles, and ossification proceeds in a ratio corresponding to the change of dentinal tissue into connective tissue cells.

When the process is ended in the entire loosening of the crown, so that it falls out with a slight touch, we see that the connective tissue of the alveolus is continuous with that of the gum, and to the naked eye the highly vascular mass of cells on which the crown rested looks like the gum deprived of its epithelium. On a close examination it looks like the healthy granulation of an ulcer or wound.

If the process has extended to completion, we find on examination of the crown that most of the dentine and considerable of the enamel has been absorbed or metamorphosed.

### HISTOLOGICAL CHANGES.

The bone corpuscles of the cement change their shape and become connective tissue cells, the latter proliferate, so that all the space is occupied with cells. The dentine tubes, also, are gradually changed into the same kind of cells as those of which they are composed before dentine tubes were formed, namely: connective tissue cells. The same changes take place within the pulp cavity, the metamorphosis commencing next to the pulp, and the latter grows in size by a transformation of dentine tubes into connective tissue cells, of which the pulp is composed, and from which the dentine tubes were originally differentiated. At the bottom and sides of the alveolus, the cells of connective tissue are gradually changed to bone corpuscles, and then the intercellular space is filled with calcareous matter, and true bone is formed.

It is difficult to decide whether the walls of the dental tubes and cement corpuscles liquify and pass by osmotic action into the connective tissue cells with which they are in contact, or whether, the differentiation of cells really takes place.

The cell multiplication which takes place in the peri cementum and pulp, looks like the former, and yet the latter would seem the more natural process. It is quite probable that both processes exist in the changes which occur. It is not likely the nuclei of the cells forming dentine are destroyed when the tubular structure takes the place of the cellular. In other tissues we find the nucleus thrust aside against the cell wall when some particular function is to be performed and cell multiplication has ceased. This takes place in bone, articular cartilage, the nails, etc. The application of some chemical agent, sometimes an acid and sometimes an alkali, will generally bring the nuclei to view under the microscope.

I have spoken of the changes, which occur, just as tho no tooth of replacement occupied the alveolus of the milk tooth, as its root is being removed. This was to simplify the process. This sometimes happens in fact, tho almost universally the tooth of replacement follows closely on to the metamorphosis of the root. All space, however, not occupied by either tooth, is filled with bone or else transitional tissue.

I have said that cellular activity is going on *within* the tooth at the same time as it progresses without. We sometimes observe that the former process outstrips the latter, and then the crown frequently falls off, leaving the roots in their alveoli unabsorbed, tho this does not hinder their removal by cell action, for it still goes on till the smallest particle of root is removed.

How happens it then that portions of milk roots are often left in the gums without absorption? It is because they are necrosed, dead. We know that frequently the whole tooth is necrosed from decay and suppuration. In this case no portion of it is absorbed, but the whole is thrown off from the system as a foreign body, either in mass or by chemical disintegration. What happens when the pulps have been destroyed and removed from milk teeth, and the cavities plugged? Absorption then takes place in the roots alone, and the crown is consequently retained longer in the jaw than when the pulp is left to act its part also.—*Missouri Dental Journal.*

---

ARTICLE VIII.

